# A New Perspective on Emerging Knowledge Translation Practices: Comment on "Sustaining Knowledge Translation Practices: A Critical Interpretive Synthesis"

**DOI:** 10.34172/ijhpm.2022.7545

**Published:** 2022-12-12

**Authors:** Anita Kothari, Jacqui Cameron

**Affiliations:** ^1^School of Health Studies, Western University, London, ON, Canada.; ^2^School of Health and Society, University of Wollongong, Wollongong, NSW, Australia.; ^3^Department of Social Work, The University of Melbourne, Parkville, VIC, Australia.

**Keywords:** Knowledge Translation, Networks, Research Co-production, Non-researcher Voices, Health Learning Systems

## Abstract

The critical interpretive synthesis by Borst and colleagues offered a new perspective on knowledge translation (KT) sustainability from the perspective of Science and Technology Studies. From our applied health services perspective, we found several interesting ideas to bring forward. First, the idea that KT sustainability includes the ongoing activation of networks led to several future research questions. Second, while not entirely a new concept, understanding how KT actors work strategically and continuously with institutional rules and regulations to sustain KT practice was noteworthy. We add to the discussion by emphasizing the importance of non-researcher voices (clinicians, administrators, policy-makers, patients, carers, public) in sustaining KT practice. We also remind readers that the health ecosystem is dynamic and interdependent, where one system level influences and is influenced by another, and that these constant adaptations suggest that understanding KT practices cannot be a one-off event but represent repeated moments for transformative learning.

## Background

 The aim of the critical interpretive synthesis by Borst et al^1^ was to identify and explain those processes, activities, and efforts in the literature that facilitate the sustaining of knowledge translation (KT) practices in health policy-making processes. Digging a little deeper, it becomes clear that essentially the authors’ focus is on how the Science and Technology Studies (STS) literature can contribute to the health policy and systems field on the premise that what KT sustainability means and how it can be improved needs further attention. The authors take a rigorous critical interpretive synthesis approach to understanding the literature in health policy and systems, and how STS can add value. At the outset, the authors note that KT needs to be re-conceptualized from an endpoint process to a dynamic process, which suggests a need to examine everyday practices. Through their analysis the authors identified three aspects of KT sustainability work: (1) translating, (2) contexting, and (3) institutionalizing KT practices. This article seems to be targeting health services researchers and policy-makers.

 This synthesis was extremely interesting, and we offer our reflections in this commentary. First, however, understanding our positionalities is necessary as our remarks are naturally influenced by the lenses we bring to our own research. The first author, a Professor in a School of Health Studies, has for the last 20 years aimed to identify the factors that influence the successful implementation of KT initiatives into healthcare decision-making (ie, practice and policy). This has involved understanding the relationship between evidence and practice/policy, including research impact, to better support the uptake and use of evidence. Most recently she has spent time understanding the processes and outcomes of integrated KT/research co-production, where those who use research findings (clinicians, administrators, policy-makers, patients, and the public) are included as research partners in the generation of evidence. The second author is a Senior Lecturer in Social Work, with a collaborative research background in alcohol and drug, mental health, and domestic violence research. She has a passion for combining evidence-based practice, KT and mixed-method research to highlight the voices of practitioners and service users as part of the research process. Thus, both of us favor applied health services and community-based research, drawing from a pragmatic philosophical tradition. We both approached this synthesis with minimal background in STS studies.

## Discussion

###  Networks

 One of the most interesting synthesis findings to us was the idea that, along with the traditional task of knowledge tailoring for dissemination, translating includes *activating networks* to bring knowledge production and knowledge utilization closer together. This aspect of KT practice draws from actor-network theory to suggest that networks are constantly being built and rebuilt between researchers, knowledge users and their environments to sustain KT practices. We note that key actors are also required to bring together unconnected groups within a network to support scaling and implementation. As an example, a domestic violence research network in Australia^2^ was able to successfully bring together a range of researchers from a cross-section of disciplines to this network using a knowledge broker. Networks, as a social context, are important for KT practices because they determine what knowledge is valued and available, and how this knowledge is negotiated against the backdrop of relationships.^3^

 An implication raised in the synthesis is that a greater focus is needed on how these connections are nurtured in relation to the everyday practice of KT sustainability work. For example, one might ask how the relationships that structure the social context create expert KT practitioners, or how the interactions bring underrepresented voices or knowledge to the work.^4^ Understanding how artefacts or objects mediate the network building and sustainable KT practice has also been raised as a noteworthy avenue of inquiry.^4^ The answers to these questions will illustrate how to develop effective and productive networks, which in turn can be used to strengthen networks as they develop over time. Several studies^5-7^ have described the carriage of time and connection among actors as a way to strengthen research networks.

###  Institutionalizing Knowledge Translation Practice

 By using the word ‘institutionalizing,’ Borst et al^1^ signal a more active position towards institutions in sustaining KT practices. Such activity is needed as institutions, ie, the rules and procedures that govern individuals, are seen as dynamic rather than steadfast. The synthesis notes that the health policy and systems literature can be augmented with accounts of how KT actors work with institutions strategically to sustain the KT practice. Instead of simply listing organizational barriers and facilitators to implementation, describing this ‘active practice’ shifts to creating a picture of *why and how* certain institutions are more conducive to particular KT innovations. Langley and Denis^8^ elaborate on this approach when discussing quality improvement initiatives in healthcare organizations. They argue that KT practices will be beneficial for some but not others in an organization, and consequently, KT actors need to negotiate with the institution to decrease the disadvantages (eg, advocating for more staff, building coalitions to create a holding space for the KT practices). Langley and Denis^8^ might suggest that sustaining a KT practice requires reframing the institution as a political system and acknowledging that simply having rigorous evidence for the practice is not enough to sustain it.

 We suggest that what is actually needed is understanding how organizations view evidence and knowledge in the first place, before any KT practice is introduced. A recent survey^5^ highlighted the considerable barriers researchers within a network encounter within their various institutions; innovative engagement mechanisms to communicate research findings were limited, and KT barriers included budget, time, capacity, limitation of models, organizational emphasis, and support. In our experience, organizations tend to be responsive to research, either as data providers or as users of research findings. But organizations need to be supported such that they can be *comprehensively engaged* in KT efforts at the outset, from knowledge generation to implementation of new KT practices. To illustrate, we found that dedicated leadership within an authorizing environment which includes understanding by leaders of time, effort and costs and benefits of knowledge utilization is required to build research capacity.^9^ KT researchers and organizations are encouraged to discuss the benefits of research through institutionalized KT practices related to patient care, organizational performance, and alignment of operations with strategic vision.^10^

###  Non-researcher Voices

 We noticed a missed opportunity, however, in that the synthesis findings did not dive deeply into the current drive for broad, planned engagement apart from naming engagement as a different site of knowledge production. There is a strong movement in applied health services to bring non-researcher voices into service delivery, research, or governance. Clinicians, practitioners, administrators, policy-makers, patients, carers and the public have local knowledge and experience that are equally important as the expertise that researchers bring to projects. The general premise is that collaboration will lead to a greater impact of treatments or research findings, leading to improved health outcomes. By inviting multiple perspectives to contribute to: optimizing service delivery (through co-design efforts, for example), influencing the research design, data collection, analysis and dissemination process, or governing tasks like priority-setting through deliberative dialogue, non-research guidance will result in outcomes or outputs that are relevant and feasible, and more likely to be applied in a sustainable way. Discussions about sustainable KT practice need to include how non-researchers are recruited, included, and supported in applied health services in an on-going way. Doing so presents an opportunity to rebalance the power of underserved voices or hidden knowledge through KT work. As Borst et al^1^ point out, it is essential to work with the context, and we propose that clinicians, administrators, policy-makers, patients, carers and members of the public are an important part of the context that healthcare organizations are paying more attention to in their daily operations.

 How these actors, especially those with lived experience and their carers, contribute to sustainable KT practices is important to understand. For example, training programmes and their development need to include a range of perspectives to increase the potential for sustainable KT practice. Our work identified the need for multiple strategies using different kinds of evidence (not just peer-reviewed but lived experience voices), especially for new and emerging populations, to contribute to collaborative capacity building.^2^ However, as noted by Banner et al^11^ there are some challenges to overcome including the nature and scope of engagement, and ensuring that engagement is beyond the tokenistic.

###  Health Learning Systems

 Applied health sciences researchers would assign a complicated relationship to KT practices, contexts and institutions that was largely absent in Borst and colleagues’ synthesis.^1^ Their resulting framework specifies that the everyday work of sustaining KT practices involves constructing amenable contexts and stable institutional climates, seemingly in a unilateral direction. Yet diverse but related applied health sciences frameworks, like socioecological theory, complex adaptive systems or health learning systems all underline the importance of interdependence among different system levels. Here, this would imply that the context and institution are not only re-negotiated and adjusted by KT actors but also that the context and institution in turn *modify* everyday KT practice. The concept of health learning systems embraces the idea of constant interactions, adaptations, and action by promoting continuous data-driven learning and transformation for improved care. The concept suggests that the systematic uncovering of KT practices, as suggested by Borst et al,^1^ ought to be an ongoing process for researchers or organizations for subsequent learning and adjustment. For example, participation in health as described by Palmer^12^ is the ‘spirit of our times’ meaning that patients are collaborating and negotiating their own healthcare through many mechanisms including treatment decision-making, online health communities/apps and as well as more formal impacts on health quality and development such as coproduction and co-design of healthcare. Thus, there is a direct link between the participation of patients and their carers in the health system and how this impacts KT practices.

## Conclusion

 Our joint interpretation of the addition of the STS literature explored by Borst et al^1^ is that KT is not an endpoint but a practice, which leads to understanding its sustainability, and that KT is a situated practice, requiring ethnographic approaches but also approaches that are open to a new ‘voice’ of actors including clinicians, administrators, policy-makers, patients, carers as well as member of the public. With any new innovation, there is the danger that the innovation will result in the mundane and compromise ongoing sustainability. Thus, we need to develop a more nuanced understanding of how this has happened. Future research needs to be explicit and ask the right questions such as: are we sharing a common language, or are we are aware of the environment in which the KT intervention will be embedded? We also need to recognize that the work of arranging contexts and influencing institutions is ongoing, and in reality, it is a small ‘p’ political practice.^8^ Thus, in retrospective studies we need to ask: what kind of political change did you need to stimulate, and how did you do this? In prospective studies need to ask^8^ who wins, who loses, and how can we get there?

## Ethical issues

 Not applicable.

## Competing interests

 Authors declare that they have no competing interests.

## Authors’ contributions

 Both authors conceptualized the work. AK wrote the first draft, and both authors contributed to its development. Both authors reviewed and approved the final version.

## References

[1] Borst RAJ, Wehrens R, Bal R. Sustaining knowledge translation practices: a critical interpretive synthesis. Int J Health Policy Manag. 2022. 10.34172/ijhpm.2022.6424. PMC10105179 35279039

[2] Cameron J, Humphreys C, Kothari A, Hegarty K (2021). Creating an action plan to advance knowledge translation in a domestic violence research network: a deliberative dialogue. Evid Policy.

[3] Oliver K, Faul MV (2018). Networks and network analysis in evidence, policy and practice. Evid Policy.

[4] Fransman J (2018). Charting a course to an emerging field of “research engagement studies”: a conceptual metasynthesis. Research for All.

[5] Cameron J, Humphreys C, Hegarty K (2021). Knowledge translation activity of a domestic violence research network: a scoping survey. Journal of Gender-Based Violence.

[6] Powell A, Davies H, Nutley S (2017). Missing in action? The role of the knowledge mobilisation literature in developing knowledge mobilisation practices. Evid Policy.

[7] Davies HT, Powell AE, Nutley SM. Mobilising Knowledge to Improve UK Health Care: Learning from Other Countries and Other Sectors–A Multimethod Mapping Study. Health Services and Delivery Research 2015;3(27). 10.3310/hsdr03270. 26110190

[8] Langley A, Denis JL (2011). Beyond evidence: the micropolitics of improvement. BMJ Qual Saf.

[9] Cameron J, Humphreys C, Kothari A, Hegarty K (2020). Exploring the knowledge translation of domestic violence research: a literature review. Health Soc Care Community.

[10] Bowen S, Graham I, Botting I. It’s Time to Talk About Our Relationship With Research. Ottawa: The Integrated Knowledge Translation Research Network; 201.

[11] Banner D, Bains M, Carroll S (2019). Patient and public engagement in integrated knowledge translation research: are we there yet?. Res InvolvEngagem.

[12] Palmer VJ (2020). The participatory zeitgeist in health care: it is time for a science of participation. J Particip Med.

